# Initial Motivations for Choosing Teaching as a Career

**DOI:** 10.3389/fpsyg.2022.842557

**Published:** 2022-06-08

**Authors:** Myriam Alvariñas-Villaverde, José Domínguez-Alonso, Lucía Pumares-Lavandeira, Iago Portela-Pino

**Affiliations:** ^1^Department of Special Didactics, Faculty of Education and Sport Sciences, University of Vigo, Vigo, Spain; ^2^Research Group on Education, Physical Activity and Health (GIES10), Galicia Sur Health Research Institute, Vigo, Spain; ^3^Department of Psychosocioeducational Analysis and Intervention, Faculty of Educational Sciences and Social Work, University of Vigo, Vigo, Spain; ^4^Department of Health Sciences, Faculty of Health Sciences, Isabel I University, Burgos, Spain

**Keywords:** perceptions, degree choice, scale validation, teaching students, career

## Abstract

The objective of the study is to verify the reliability and validity of the Factors Influencing Teaching Choice, as well as to determine the teaching of Early childhood and Primary education students’ motivations and perceptions for the choice of this degree. The sample consisted of 262 student teachers aged between 18 and 27 (83.2% female and 16.8% male). According to degree, 54.2% of the participants were enrolled in Early Childhood Education and 45.8% in Primary Education. The instrument used in the study was the Factors Influencing Teaching Choice, FIT-Choice Scale, adapted to Spanish by Gratacós and López-Jurado. Results evidenced the high reliability and validity of the scale that is similar to the original model, presenting 12 motivational factors and 6 perceptions. The main reasons that lead to choose teaching profession, despite gender differences, are: having influence in the educational children’s process, the intrinsic value of the qualification, the fact that this profession contributes socially, or working with children. Also, students consider that teaching is a demanding profession and they are satisfied with the degree choice. The most important factors influencing the choice of education degree are intrinsic motivations and perceptions as the vocation, in contrast to external reasons such as economic incentives or social recognition.

## Introduction

Today’s society needs highly educated and motivated teachers. The teacher training students’ motivations for the choice of a degree and during their professional performance contribute to increasing the quality of the teaching-learning systems. Fokkens-Bruinsma and Canrinus (2014) have indicated that the reasons which motivate the choice of a degree program are fundamental to teachers’ commitment to their professional performance.

Motivation plays an important role in decision making because impulses, desires, expectations, evaluations, and other motivational tendencies are determining factors of the degree of commitment to action. For this reason, and given that motivation has been considered one of the factors that best explain human behavior in different contexts (Burgueño et al., 2017), since teachers’ motivations are remarkably stable over time (Praetorius et al., 2017), teacher training students’ motivation is a key aspect to be analyzed. If an analysis focuses on improving the selection and training of future teachers, it should be based on knowledge of their motivations (Watt and Richardson, 2008; Gratacós and López-Jurado, 2016; Watt et al., 2017). In addition, in order to give prestige to the teaching profession, it would be of enormous importance to find out the Early Childhood and Primary Education teacher training students’ perceptions, as well as the factors that affect the choice of these university studies (Gratacós and López-Jurado, 2016).

The motivation is key so that future teachers could choose this career path and stay on it (Fokkens-Bruinsma and Canrinus, 2012). While it is true that, at first, our intrinsic motivation and our expectations of achievement determine our choices, from that point on, the achievement of our goal will have to do with volitional processes, with the firm intention or purpose of achieving our objective (Barberá-Heredia, 2002). In addition, according to the expectancy-value theory, the achievement expectations are the main reasons for predicting academic choices and behaviors (Watt and Richardson, 2007). All of this highlights the importance of maintaining students’ levels of motivation during their initial and continuous training.

On the other hand, knowing the motivations that lead to the choice of degrees in Education would allow us to carry out a more effective career guidance to the students before going to University. In addition, the design of the university’s initial training could be improved to prevent students from losing interest and reconsidering their career path (Eren and Tezel, 2010; Watt et al., 2012; Zúñiga et al., 2015; Burgueño et al., 2017). Therefore, the establishment of effective policies aimed at strengthening the teaching profession and ensuring the attraction of good candidates requires the identification and understanding of factors that may attract or discourage candidates from choosing Education studies (Eren and Tezel, 2010; Gratacós and López-Jurado, 2016; Said-Hung et al., 2017). Currently, in Spain, the analysis is focused on the model of access to the teaching profession, as well as the criteria for training throughout the professional life.

It is thus of vital importance to have valid and reliable instruments that allow for evaluating the motivational factors that influence the choice of the teaching profession. The FIT-Choice Scale (Watt and Richardson, 2007) is based on the expectancy-value motivational theory, a social cognitive theory of motivation which recognizes that students have values to perform school tasks, as well as success expectations, which explains the decisions they make. For this reason, the scale classifies the determining factors of motivation into four: Self-perception, value of the task, fallback career, and expectations and beliefs about the profession (Watt and Richardson, 2007; Eren and Tezel, 2010; Klassen et al., 2011; Berger and D’Ascoli, 2012a; Watt et al., 2012; Gratacós and López-Jurado, 2016; Said-Hung et al., 2017).

The first component of the FIT-Choice Scale is self-perception, which refers to the perceived ability to teach. The literature on this subject has pointed out that this perception is fundamental in the motivation to teach, since individuals choose occupations for which they think they have the right skills, tending not to take jobs that do not fit their skills. The second component is the value of the task, which is divided into three types: The intrinsic value of the career refers to the interest in the profession; the value of personal utility is represented by job stability, labor mobility, and work-life balance; and, finally, the value of social utility is divided into children’s future, social equity, social contribution, and work with children/youth. The third component is the fallback career. This refers to the choice of Education studies when the first option was not possible. The fourth component refers to the expectations and beliefs about the profession, which are divided into professional demands, and professional satisfaction. The professional demands are subdivided into a demanding degree program and demanding profession, while professional satisfaction is subdivided into social status and salary.

Finally, the Spanish version of the FIT-Choice Scale (Gratacós and López-Jurado, 2016) has adequate internal consistency in the global scales related to motivations and perceptions. However, this scale was also validated and translated into different languages, used in countries such as Australia (Watt and Richardson, 2007), United States (Lawver and Torres, 2011), the Netherlands (Fokkens-Bruinsma and Canrinus, 2012) Australia, United States, and Germany (König and Rothland, 2012), Germany and Netherlands (Watt et al., 2012), Turkey (Eren and Tezel, 2010; Kılınç et al., 2012; Topkaya and Uztosun, 2012), Switzerland (Berger and D’Ascoli, 2012b), Canada and Omán (Klassen et al., 2011), Croatia (Jugović et al., 2012), Finland (Goller et al., 2019), China (Lin et al., 2012), Indonesia (Suryani et al., 2016), Spain (Gratacós and López-Jurado, 2016), Colombia (Said-Hung et al., 2017), or Ghana (Salifu et al., 2018).

Supported in previous studies, the reasons that can lead to considering being a teacher focus primarily on material aspects, such as job stability, professional aspects, such as a taste for certain subjects, or altruistic aspects, such as the responsibility toward children (Fokkens-Bruinsma and Canrinus, 2012). Authors such as Bertomeu et al. (2007) have pointed out the pleasure of teaching and working with children as main reasons. Following the same line, Avendaño and González (2012) have indicated as reasons to choose the teaching career the pleasure of teaching, the role of education in society, and the taste for the subjects taught. In any case, extrinsic factors also have an impact, such as professional stability, prestige associated with the teaching profession and the opportunities to acquire valuable skills that can be transferred to other professions, including proficiency in English for students doing training courses abroad (Gao and Trent, 2009).

The increased quality of the educational system involves attracting the best teachers, which is why it is very important to understand the motivations and perceptions that lead university students to choose degrees in education.

In this study, the motivations of teacher-training students for teaching and their perceptions of the teaching profession for choosing a teaching degree have been explored. Furthermore, the psychometric scale variables in this collective have been calculated.

## Materials and Methods

### Participants

The probabilistic and intentional sample was made up of 262 teacher training students aged between 18 and 27 years (average age: 21.96 ± 3.73). In terms of gender, there is a predominance of women (83.2%) compared to men (16.8%), as is usual in the degree programs under study. In addition, according to the degree, 54.2% of the participants were enrolled in the Early Childhood Education degree, and 45.8% were enrolled in the Primary Education degree. Finally, 15.6% were enrolled in the first academic year, 23.3% in the second, 30.5% in the third, and 30.5% in the fourth year. The context of the study was the University of Vigo (Galicia, Spain) and the Faculty of Education and Sport Sciences, where the aforementioned degrees are taught. No previous university degree is required for admission to these courses, but a minimum grade in secondary education is required.

### Instrument

To carry out this study, the measurement scale of the factors that affect the choice of Education Studies (Factors Influencing Teaching Choice, FIT-Choice Scale) was used, adapted to Spanish by Gratacós and López-Jurado (2016). As indicated in this research, the FIT-Choice scale showed adequate levels of reliability and factorial validity in line with the original version and with adaptations made in other languages and validated with samples from other countries. Furthermore, the internal consistency of the subscales was adequate; Cronbach’s a values for the global scales of motivations and perceptions were 0.88 and 0.69, respectively. This scale is divided into three parts: Influencing factors (38 items), beliefs about education (14 items), and the decision to be a teacher (6 items). The items contained in each of these parts have to be restructured when analyzing the data (according to the authors’ guidelines), so that they could correspond to the previously indicated components and factors. Moreover, these items are written as statements that are assessed with a Likert-type scale ranging from 1 (not important) to 7 (very important).

### Procedure

The questionnaire was administered collectively to university students during regular school hours. They were contacted in several regular classes of their degree program. After communicating the appropriate instructions and once the informed consent form was signed, all students voluntarily completed the requested information. The average time of application of the instrument was 15–20 min. The study was conducted according to the ethical standards established by the Declaration of Helsinki (1975).

### Data Analysis

First, given the sensitivity of the Chi-square statistic to the sample size, other fit indices have been used: χ^2^/df test (values lower than 3 are considered indicators of acceptable fit of the model); absolute Goodness of Fit Index (GFI ≥ 0.90), Standardized Root Mean Residual (SRMR ≤ 0.06), Root Mean Square of Approximation (RMSEA ≤ 0.08); Normed Fit Indices (NFI ≥ 0.90), Comparative Fit Index (CFI ≥ 0.90), and Adjusted Goodness of Fit Index (AGFI ≥ 0.90) (Brown, 2006; Lévy and Varela, 2006; Arbuckle, 2009; Schweizer, 2010). Finally, the internal consistency of the factors was assessed using the Cronbach’s alpha, following the recommendations of George and Mallery (2003) (α > 0.9 excellent, α > 0.8 good, α > 0.7 acceptable).

The mean and standard deviation of each motivational and perceptual factor were also calculated. Next, to obtain the results on the main effects of the interaction between the different variables, the Student’s *t*-test for independent samples was carried out. The effect size (Cohen’s d) was also calculated: values between 0.2 and 0.3 indicate a small effect, around 0.5 a medium effect, and higher than 0.8 a large effect.

All analyses were performed with the SPSS v. 23 statistical package (IBM Corp, 2013) and the AMOS v. 23 package, given that a relationship is statistically significant when *p* < 0.05.

## Results

### Analysis of the Factorial Structure

To test the factorial structure of the FIT-Choice Scale, the 18-factor model was established as a hypothetical starting model (12 motivational factors: working with children, improving social equity, influencing children’s future, making a social contribution, job stability, labor mobility, work-life balance, intrinsic value of the career, perceived ability, previous teaching/learning experiences, social influences, and fallback career; and six perception factors: demanding degree program, demanding profession, salary, social status, social dissuasion, and satisfaction with choice) validated by Gratacós and López-Jurado (2016).

The results of the scale model allow, *a priori*, to observe a factor saturation of the item (< 0.40) in our sample, and an error associated to the variance (> 0.80), confirming that the elimination of any item is not necessary. Thus, the estimation of a model with 18 factors and 58 items using the maximum likelihood (ML) method obtained a good fit to the data in the two subgroups (motivation and perception) (Table 1).

**TABLE 1:** Adjustment indices of the FIT-choice scale.

MODEL	χ^2^/df	GFI	SRMR	NFI	CFI	AGFI	RMSEA
Motivation	1.19	0.898	0.060	0.861	0.901	0.836	0.059
Perception	1.55	0.924	0.057	0.918	0.963	0.904	0.046

*For all of the Chi-square values, p < 0.001.*

In addition, it shows a good internal consistency, with a Cronbach’s alpha of 0.886 for the total scale (α = 0.870 in motivational factors and α = 0.735 in perceptual factors).

### Descriptive and Inferential Analysis of the Motivational Factors That Influence the Choice of a Degree in Education

According to the results (Table 2), it was observed that among the main reasons that led to the choice of a degree in education are: influencing children’s future (*M* = 18.85), intrinsic value of the career (*M* = 18.64), making a social contribution (*M* = 18.03), or working with children (*M* = 18.02). On the other hand, social influences (*M* = 12.86), job stability (*M* = 12.58), labor mobility (*M* = 10.68), and being a fallback career (*M* = 5.31) are among the least influential reasons.

**TABLE 2:** Means and standard deviations of motivational factors.

Order	Motivational Factors	*M*	*SD*
1.	Influencing children’s future	18.85	2.25
2.	Intrinsic value of the career	18.64	2.65
3.	Making a social contribution	18.03	2.32
4.	Working with children	18.02	3.79
5.	Improving social equity	17.59	2.98
6.	Perceived ability	17.51	2.58
7.	Work-life balance	17.50	7.51
8.	Previous teaching/learning experiences	15.82	4.78
9.	Social influences	12.86	5.67
10.	Job stability	12.58	4.52
11.	Labor mobility	10.68	4.03
12.	Fallback career	5.31	3.72

*Minimum = 1, Maximum = 7, M = mean, and SD = standard deviation.*

The results of the Student’s *t*-test for independent samples (Table 3) obtained between the motivation to choose a degree in education according to gender (men and women) showed significant differences in the following factors: working with children [*t*(1, 260) = 16.28; *p* < 0.001; η^2^ = 0.630], being higher in women; influencing children’s future [*t*(1, 260) = 19.14; *p* < 0.001; η^2^ = 0.664], being higher in women; making a social contribution [*t*(1, 260) = 4.35; *p* < 0.05; η^2^ = 0.333], being higher in women; job stability [*t*(1, 260) = 5.47; *p* < 0.05; η^2^ = 0.426], being higher in men; labor mobility [*t*(1, 260) = 7.06; *p* < 0.01; η^2^ = 0.445], being higher in men; work-life balance [*t*(1, 260) = 11.43; *p* < 0.01; η^2^ = 0.589], being higher in men; intrinsic value of the career [*t*(1, 260) = 44.54; *p* < 0.001; η^2^ = 0.965], being higher in women; perceived ability [*t*(1, 260) = 15.68; *p* < 0.001; η^2^ = 0.611], being higher in women, and fallback career [*t*(1, 260) = 34.26; *p* < 0.001; η^2^ = 0.812], being higher in men. The effect size varied between medium (η^2^ = 0.333) and very large (η^2^ = 0.965). There were no significant differences in motivational factors: Improving social equity [*t*(1, 260) = 3.79; *p* > 0.05; η^2^ = 0.327]; previous teaching/learning experiences [*t*(1, 260) = 1.29; *p* > 0.05; η^2^ = 0.192]; and social influences [*t*(1, 260) = 187; *p* > 0.05; η^2^ = 0.077]. Therefore, in terms of motivation, the data confirmed a greater presence of women in working with children, influencing children’s future, making a social contribution, intrinsic value of the career, and perceived ability. Men are more motivated by factors such as job stability, labor mobility, work-life balance, and as a fallback career.

**TABLE 3:** Means, standard deviations, Student’s *t*-test, and effect size (η^2^) of the reasons according to gender.

Reason	Female (*n* = 218)	Male (*n* = 44)	*t* (1, 260)	*p*	η^2^	Prevalence
		
	*M* (*SD*)	*M* (*SD*)				
Working with children	18.43 (3.56)	15.97 (4.22)	16.28	0.000	0.630	> Women
Social equity	17.74 (3.01)	16.79 (2.79)	3.79	0.052	0.327	ns
Children’s future	19.12 (2.07)	17.54 (2.65)	19.14	0.000	0.664	> Women
Social contribution	18.16 (2.26)	17.36 (2.53)	4.35	0.038	0.333	> Women
Job stability	12.28 (4.66)	14.02 (3.40)	5.47	0.020	0.426	> Men
Labor mobility	10.38 (4.01)	12.13 (3.86)	7.06	0.008	0.445	> Men
Work-life balance	16.81 (7.53)	20.93 (6.41)	11.43	0.001	0.589	> Men
Intrinsic value	19.09 (2.25)	16.38 (3.27)	44.54	0.000	0.965	> Women
Perceived ability	17.78 (2.42)	16.13 (2.95)	15.68	0.000	0.611	> Women
T/L experiences	15.96 (4.82)	15.06 (4.55)	1.29	0.256	0.192	ns
Social influences	12.79 (5.86)	13.20 (4.71)	0.187	0.666	0.077	ns
Fallback career	4.74 (3.12)	8.13 (5.01)	34.26	0.000	0.812	> Men

*Ns, non-significant.*

### Descriptive and Inferential Analysis of the Perceptions of Choice of a Degree in Education

Focusing on students’ perceptions of choice of a degree in education (Table 4), the fact of being a demanding profession (*M* = 19.11) and satisfaction with choice (*M* = 19.02) stood out. However, social dissuasion (*M* = 9.79) and salary (*M* = 6.64) presented the lowest means.

**TABLE 4:** Means and standard deviations of perceptions.

Order	Perceptual factors	*M*	*SD*
1.	Demanding profession	19.11	1.98
2.	Satisfaction with choice	19.02	2.47
3.	Demanding degree program	17.58	2.92
4.	Social status	15.81	6.03
5.	Social dissuasion	9.79	4.94
6.	Salary	6.64	2.80

*Minimum = 1, Maximum = 7, M = Mean, SD = Standard Deviation.*

Finally, the results of the Student’s *t*-test for independent samples (Table 5) obtained for students’ perceptions of choice of degrees in education according to gender (men and women) showed significant differences in the following factors: demanding degree program [*t*_(1_, _260)_ = 4.30; *p* < 0.05; η^2^ = 0.324], being higher in women; demanding profession [*t*_(1_, _260)_ = 12.13; *p* < 0.01; η^2^ = 0.530], being higher in women; salary [*t*_(1_, _260)_ = 4.71; *p* < 0.05; η^2^ = 0.359], being higher in men; social status [*t*_(1_, _260)_ = 18.75; *p* < 0.001; η^2^ = 0.692], being higher in men; and, satisfaction with choice [*t*_(1_, _260)_ = 37.78; *p* < 0.001; η^2^ = 0.806], being higher in women.

**TABLE 5:** Means, standard deviations, analysis of variance, and effect size (η^2^) of the perceptions according to gender.

Perceptions	Women (*n* = 218)	Men (*n* = 44)	*t* (1, 260)	*p*	η^2^	Prevalence
		
	*M* (*SD*)	*M* (*SD*)				
Demanding degree program	17.74 (2.83)	16.75 (3.25)	4.30	0.039	0.324	>Women
Demanding profession	19.29 (1.85)	18.18 (2.31)	12.13	0.001	0.530	> Women
Salary	6.47 (2.78)	7.47 (2.79)	4.71	0.031	0.359	> Men
Social status	15.11 (5.73)	19.29 (6.34)	18.75	0.000	0.692	> Men
Social dissuasion	9.72 (5.07)	10.11 (4.31)	0.220	0.639	0.082	ns
Satisfaction with choice	19.42 (1.94)	17.06 (3.66)	37.78	0.000	0.806	> Women

*Ns, non-significant.*

The effect size varied between medium (η^2^ = 0.324) and large (η^2^ = 0.806). There were no significant differences in the social dissuasion factor [*t*_(1_, _260)_ = 0.22; *p* > 0.05; η^2^ = 0.082]. Thus, it was confirmed that women had a greater perception of the demands as a degree program/profession, and satisfaction with their choice, while men in reference to salary and social status.

## Discussion and Conclusion

The aim of this study was to analyze the reliability and validity of the FIT-Choice scale and to determine the motivations and perceptions that have led students to choose a degree in Early Childhood or Primary Education, given the positive relationship between motivational constructions and professional learning (Durksen et al., 2017).

The results obtained in the confirmatory analysis follow the patterns of a previous study conducted by Gratacós and López-Jurado (2016). Considering the factorial structure of the FIT-Choice Scale, the results confirmed the raised hypothesis, since the model containing 12 motivational and six perceptual factors obtained an adequate fit to the data. This allows us to conclude that the factorial model of the Spanish version of the FIT-Choice Scale is adequately fit to the original theoretical model, containing 12 motivational factors (working with children, improving social equity, influencing children’s future, making a social contribution, job stability, labor mobility, work-life balance, intrinsic value of the career, perceived ability, previous teaching/learning experiences, social influences, and fallback career) and six perception factors (demanding degree program, demanding profession, salary, social status, social dissuasion, and satisfaction with choice) in the teaching vocation.

The teaching profession no longer appeals to the future generations in Spain or in the countries belonging to the Organization for Economic Co-operation and Development (OECD, 2019). The report Teachers and School Leaders as Lifelong Learners, based on the OECD Teaching and Learning International Survey (TALIS), highlights that to guarantee young people receive the skills they need to thrive in the labor market, the teaching profession needs to attract the best and most brilliant students.

More than 80% of the teacher-training students in our sample are women, relatively young, and with a high average entry grade. We must bear in mind that according to the aforementioned report, women (68%) are still the majority in this profession, with the exception of Japan (42%). In some contexts, despite the fact that teaching is even more popular as a first-choice degree among women (Yüce et al., 2013), this does not always match professional activity; furthermore, few reach managerial positions (47%) (OECD, 2019).

The lower rate of men in the teaching profession, above all in infant and primary education, is still a topic of debate. Parr et al. (2008) highlight that it is a problem in most countries where poor performance among boys, the lack of male role models, and gender equality are a concern. In Ontario, these authors claim that men represent less than a tenth of primary teachers and these numbers are falling.

On the other hand, the different motivational factors that lead students to choose degrees in education are listed by descending order: having influence on children’s educational process (altruistic motivation), the intrinsic value of the career (intrinsic motivation), the fact that this profession contributes socially, or working with children (altruistic motivation). In the same way, the factors of least influence in the choice of this career are related to extrinsic motivation: social influence, job stability, or labor mobility.

Similar results were found in the study carried out by Said-Hung et al. (2017) in Colombia. This study indicated that altruistic reasons (influencing children/adolescents’ future or making a social contribution) and intrinsic reasons (previous teaching/learning experiences) were highly valued by the students surveyed. However, the extrinsic reasons, related to salary or work-life balance, had a lower value, except for the factor referring to job stability. Also in the TALIS report, 90% of teachers mention the chance to contribute toward the development of children and society as one of their main motivations to join the profession. Similarly, Watt et al. (2017) note that people who choose teaching as a career are motivated by a complex interplay of factors rooted within communities and cultural expectations, but that they have in common a desire to undertake work that supports the consolidation of a better society.

Fokkens-Bruinsma and Canrinus (2012) also emphasize that the main motivation to become a teacher is self-perception of teaching-related abilities and highlight other motivations such as working with children, previous teaching and learning experiences, and time to spend with their families, as well as satisfaction with the choice of teaching and perceived work demand. The students who are already in the last year of their degree also highlight social influences as motivations. In this same line of thought, Hongjie (2016) has also reached the conclusion that the biggest professional motivation is having time to spend with the family, wanting to contribute to society, the working environment, intrinsic value of teaching, and self-perception.

In our study, results are not conclusive regarding the academic year, and first-year students have more extrinsic motivation while third-year students are more motivated by professional demands. Nevertheless, in the Zehir-Topkaya and Uztosun (2012) study, there were no statistical differences between the professional motivations of first- and fourth-year students.

In other words, it is a combination of different reasons (intrinsic, extrinsic, and altruistic), emphasizing that beliefs about teaching are eclectic and previous experiences influence them (Thomson et al., 2012). In fact, normally job satisfaction increases if the students have received practical training during their academic stages and are usually more critical with the lack of coherence during their teaching activities (Roness and Smith, 2009).

In addition, the study carried out by Klassen et al. (2011), performing a comparison between the reality of Canada and that of Oman, revealed that, despite cultural differences, students chose to study degrees in education for the same reasons. These mainly refer to the perceived ability, the intrinsic value of the career (intrinsic motivation), and the reasons for personal utility (extrinsic motivation). No differences were found between countries in terms of choice of education studies in the research carried out by Watt et al. (2012) in Australia, United States, Germany, and Norway. The authors found that the most valued reasons for the choice of a teaching career were the intrinsic value of the career (intrinsic motivation), the perceived ability, the desire to make a social contribution (altruistic motivation), working with children/adolescents (altruistic motivation), and having had previous teaching-learning experiences.

In the comparative study between the United States and China, Lin et al. (2012) notes that the participants were also motivated to go into teaching because of social usefulness; although the trainee teachers in the United States have significantly higher motivations in the values of social usefulness, teaching skills, intrinsic values of the profession and previous teaching experiences and learning, and professional motivations are lower. The participants from the United States are more satisfied with their career choice. In a recent study conducted in Nigeria (Akpochafo, 2020) social utility values were the most influential in university students’ choice of teaching as a career. In Turkey, the Kılınç et al. (2012) study also highlights altruistic social usefulness values as the most influential motivations of the future teachers followed by the desire for a steady job. Intrinsic values and self-perceived teaching abilities were the most highly rated options.

In the De Cooman et al. (2007) study where working teachers and recently graduated teachers are compared, the conclusion is that the former consider intrinsic, altruistic, and interpersonal factors as strong specific motivators of their work. In the Marušić-Jablanović and Vraèar (2019) study, the degree choice of future teachers is also based on factors such as intrinsic values, social usefulness, self-perceived abilities, and previous teaching experiences and learning. Nevertheless, they also noted reasons such as work security and having time to spend with their families.

In addition to the aforementioned, there is evidence that indicate that the motivations for the choice of the teaching profession vary according to gender. Thus, the motivational factors of greatest impact for women include the work and influence on children’s life or choose a profession that contributes socially, while men are inclined toward job stability, labor mobility, or work-life balance. Therefore, women are more motivated by altruistic and intrinsic motivation and professional demands. Men are more motivated extrinsically and by professional gains. It has also been proven in Bruinsma and Jansen (2010) and Yüce et al. (2013) studies that altruistic motivations are greater in women and extrinsic motivations are more common among men. Similarly, the work of Silvestre et al. (2020) shows that female students are more motivated to work with children and adolescents. With regard to teachers of English, Zehir-Topkaya and Uztosun (2012) reached similar conclusions. All trainee teachers have greater intrinsic motivation and are socially motivated, but the male participants rate more highly in motivations such as work security and employment prospects.

Teaching is the first choice degree for most of the students interviewed despite the fact that, as Sinclair (2008) highlights, the demands on teachers are very high and there are many new professions to choose from. In the TALIS report, teaching is also the first choice for two out of every three teachers in the OCDE countries; however, it is the first choice for only 59% of male teachers, compared to 70% of female teachers. It should be noted that the choice of teaching as a fallback career occurs to a greater extent in men than in women. Therefore, there is no consensual approach pointing to the fact that the education degrees are chosen as a fallback career, this being the least valued factor most of the time (Said-Hung et al., 2017). Moreover, although the studies do not differentiate the choice of an education degree according to gender, making it difficult to contrast our results (Klassen et al., 2011; Watt et al., 2012; Said-Hung et al., 2017), López-Sáez et al. (2004) pointed out that women’s motivation in the choice of a career is linked to vocation and helping others, while men choose it for reasons such as salary, prestige, and professional status or projection.

Regarding the perceptual factors, the participants in this study consider that teaching is a demanding profession and they are satisfied with choosing this career, social dissuasion and salary being the factors that obtained the lowest means. There are gender differences in this case too, since women’s perceptions are greater in terms of demanding degree programs, demanding profession, and satisfaction with choice, while men’s obtained the highest score in the salary, and social status factors. The study by Silvestre et al. (2020) also indicates differences in perceptual factors, with higher ratings for girls. The research conducted by Said-Hung et al. (2017) also showed that the factors related to perceptions that obtained the highest score were the views regarding the demands of the profession and satisfaction with the choice of this career. However, the factors related to salary, social status, and social dissuasion obtained the lowest scores.

Regarding pay, the Yuan et al. (2013) study checked whether financial incentives motivated teachers to improve the performance of their students and showed that for most teachers it was not a motivating element. We must take into account that the difficulties to recruit and hold on to teachers are related to the work load and pay of the teacher, as well as to problem students and the low status of the profession (Kyriacou et al., 2003).

If we take into account this contextual aspect, in studies such as the one by Goller et al. (2019), where motivations and perceptions are also studied, it is evident that although the motivations for teaching in Finland and Germany are similar, the teaching profession is viewed differently. Likewise, Watt et al. (2012) state that the motivations for teaching are similar, while the perceptions of the teaching profession tend to reflect the cultural differences of each country.

Therefore, we can **conclude** that the motivations of the teacher training students to choose the profession are focused primarily on the intrinsic value and social usefulness of the latter, as it enables people to work with children, have an influence in their future, and contribute to society. Second, intrinsic motives of a personal nature are valued, as self-perceived abilities or work-life balance. Likewise, the most highly valued perceptions are the demands of the profession and the satisfaction with the choice.

On the other hand, since teaching is a highly feminized profession (Bravo et al., 2006) the results show that the factors that most influence the choice of degrees in education are intrinsic motivations and perceptions, such as vocation, in contrast to external reasons, such as economic stimuli or social recognition (Franco-López et al., 2015). It should be noted that the differences found between men and women may derive from gender stereotypes, which underlie motivations and perceptions of the choice of a teacher training degree.

As limits of the research, the fact that the data were collected cross-sectionally and by means of self-reporting stands out. This type of work has biases that can influence the generalizability of the data, so its results and conclusions are affected by contextual and cultural issues. It would be interesting to continue future research by delving into the subject from a qualitative point of view, in order to obtain a deeper and more specific knowledge of the situation.

With this study, the aim is to make a useful contribution to scientific proof of teacher training, as despite the abundance of literature on teaching motivation, there is little evidence on motivations, beliefs, or experiences for the choice of this profession. We consider that getting to know the motivational characteristics of this collective is important (Gratacós and López-Jurado, 2016) to design quality educational policies and teaching practices. This will allow us to carry out future studies in depth with the goal of attracting, retaining, and educating the next generation of teachers, offering new ideas on the training process and curriculum development.

## Data Availability Statement

The data are not publicly available due to confidentiality reasons. Requests to access the datasets should be directed to MA-V (myalva@uvigo.es).

## Ethics Statement

Ethical review and approval were not required for the study on human participants in accordance with the local legislation and institutional requirements. Written informed consent to participate in this study was provided by the participants’ legal guardian/next of kin.

## Author Contributions

IP-P and MA-V: theoretical framework. JD-A: research design. LP-L: data collection. IP-P and JD-A: data analysis. IP-P and MA-V: writing-preparation of the original draft. IP-P and MA-V: final writing and revisions. All authors reviewed and accepted the final manuscript.

## Conflict of Interest

The authors declare that the research was conducted in the absence of any commercial or financial relationships that could be construed as a potential conflict of interest.

## Publisher’s Note

All claims expressed in this article are solely those of the authors and do not necessarily represent those of their affiliated organizations, or those of the publisher, the editors and the reviewers. Any product that may be evaluated in this article, or claim that may be made by its manufacturer, is not guaranteed or endorsed by the publisher.
